# Expanded functionality, increased accuracy, and enhanced speed in the *de novo* genotyping-by-sequencing pipeline GBS-SNP-CROP

**DOI:** 10.1093/bioinformatics/bty873

**Published:** 2018-10-15

**Authors:** Arthur T O Melo, Iago Hale

**Affiliations:** Department of Agriculture, Nutrition, and Food Systems, University of New Hampshire, Durham, NH, USA

## Abstract

**Summary:**

GBS-SNP-CROP is a bioinformatics pipeline originally developed to support the cost-effective genome-wide characterization of plant genetic resources through paired-end genotyping-by-sequencing (GBS), particularly in the absence of a reference genome. Since its 2016 release, the pipeline’s functionality has greatly expanded, its computational efficiency has improved, and its applicability to a broad set of genomic studies for both plants and animals has been demonstrated. This note details the suite of improvements to date, as realized in GBS-SNP-CROP v.4.0, with specific attention paid to a new integrated metric that facilitates reliable variant identification despite the complications of homologs. Using the new *de novo* GBS read simulator GBS-Pacecar, also introduced in this note, results show an improvement in overall pipeline accuracy from 66% (v.1.0) to 84% (v.4.0), with a time saving of ∼70%. Both GBS-SNP-CROP versions significantly outperform TASSEL-UNEAK; and v.4.0 resolves the issue of non-overlapping variant calls observed between UNEAK and v.1.0.

**Availability and implementation:**

GBS-SNP-CROP source code and user manual are available at https://github.com/halelab/GBS-SNP-CROP. The GBS read simulator GBS-Pacecar is available at https://github.com/halelab/GBS-Pacecar.

**Supplementary information:**

Supplementary data are available at *Bioinformatics* online.

## 1 Introduction

The GBS-SNP-Calling Reference Optional Pipeline (GBS-SNP-CROP) is an open-source pipeline that integrates custom parsing and filtering procedures with well-known, vetted bioinformatic tools, giving users full readable access to all intermediate files. Initially designed for paired-end reads, GBS-SNP-CROP employs a strategy of variant calling based on both within-individual and across-population patterns of polymorphism to identify and distinguish high-confidence variants from both sequencing and PCR errors, whether or not a reference genome is available. In the latter case, the pipeline uses a read-clustering strategy to build a so-called Mock Reference (MR) of consensus GBS fragments for use in downstream alignment, variant calling, and genotyping (Melo *et al.*, 2016).

As a reference-optional (or *de novo*) pipeline, GBS-SNP-CROP has proven useful to breeders of under-researched crop species for which the lack of a reference genome presented a barrier to the efficient use of GBS data (Cheng *et al.*, 2017; Hale *et al.*, 2018; Melo *et al.*, 2017; Sogbohossou *et al.*, 2018; Wang *et al.*, 2017). The pipeline has facilitated studies of genetic diversity and population structure for natural populations of both plants (Arredondo *et al.*, 2018; Bartaula *et al.*, 2018; Sunseri *et al.*, 2018) and animals (Drury *et al.*, 2017; Xu *et al.*, 2017) and has successfully supported molecular breeding analyses in a variety of crop and non-crop plant species (Adhikari *et al.*, 2018; Chung *et al.*, 2018; Holloway *et al.*, 2018; Munjal *et al.*, 2017). In presenting GBS-SNP-CROP v.4.0, this note describes the expanded functionality and enhanced performance of the pipeline relative to its original version.

## 2 Enhanced functionality and performance

Since its initial release, GBS-SNP-CROP has been updated with a suite of functional enhancements. Specifically, the pipeline now: (i) accommodates both single-end and paired-end reads; (ii) identifies and calls bi-allelic indels as well as SNPs; (iii) improves overall memory usage and employs parallelization to substantially reduce computation time; (iv) supports conversion of the final genotyping matrix into standard Variant Call Format (VCF); (v) creates a set of comprehensive variant description files to support user decision-making in the application of subsequent filters; (vi) enables the identification and filtering of likely paralogous/duplicated loci, based on the strategy of McKinney *et al.* 2017; and (vii) facilitates ploidy inference based on individual distribution of allele depth ratios at heterozygous loci, as proposed by Yoshida *et al.*, 2013 (Supplementary Fig. S1).

### 2.1 Computation time and data usage

A notable feature of GBS-SNP-CROP v.4.0 is the parallelization of raw read and genotype-specific mpileup file parsing via the Parallel::ForkManager CPAN module. On a Unix workstation with 16 GB RAM and a 2.6 GHz Dual Intel processor, v.4.0 requires ∼14 minutes to parse 1 Gb of raw sequence data, compared to ∼49 minutes under the initial version. On the same machine, v.4.0 completes a full analysis of 55 Gb of 150-bp paired-end data (a population of 96 *Berberis* × *ottawensis* hybrids) in ∼13 h, compared to the ∼45 h required by v.1.0, a time saving of 71%. A similar time saving (67%) was observed using reads generated by GBS-Pacecar (Table 1), a *de novo* GBS read simulator available at https://github.com/halelab/GBS-Pacecar.

**Table 1.** Comparative summary of GBS-SNP-CROP v.4.0 performance, based on a set of simulated data from GBS-Pacecar

Pipeline^a^	MR geno^b^	Time (min)^c^	Variants called^d^	Type I error^e^	Type II error^f^	Accuracy^g^
UNEAK	NA	8.5	2642	0.9%	92.5%	7.5%
GSC v.1.0	1	370.8	23 395	1.3%	34.1%	65.4%
GSC v.4.0	1	121.7	29 738	0.6%	15.6%	84.0%
	5	156.9	26 885	0.6%	23.6%	76.0%
	10	171.5	26 854	0.5%	23.7%	76.1%
	15	179.1	26 897	0.5%	23.6%	76.1%
	20	183.0	26 892	0.5%	23.6%	76.1%
	25	163.2	26 901	0.5%	23.5%	76.2%

*Note*: In total, 25 000 SNPs and 10 000 indels were simulated across a genomic space of 100 000 GBS fragments. A total of 60 002 165 single-end reads were simulated for a population of 25 individuals (average of 2.4 million reads per genotype), with a sequencing error rate of 1.1%. See Supplementary Table S1 for more details

a UNEAK = TASSEL-UNEAK; GSC = GBS-SNP-CROP.

b The number of genotypes used for mock reference (MR) assembly.

c Computation time (minutes) required to run the full analysis on a Unix workstation with 16 GB RAM and a 2.6 GHz Dual Intel processor.

d Number of variants called by a pipeline (Note: a total of 35 000 variants were simulated, consisting of 25 000 SNPs and 10 000 indels).

e Percentage of called variants that could not be validated (false positives).

f Percentage of true, simulated variants that were not detected by the pipeline.

g Overall accuracy: 100 * [number of validated variants/(total number of simulated variants + number of non-validated variants)].

Because of its superior speed and clustering capabilities, including fewer ‘missed’ alignments and chimeric centroids, Vsearch (Rognes *et al.*, 2016) is now called by GBS-SNP-CROP rather than Usearch (Edgar, 2010) for MR construction (Step 4). While this change effectively lifts Usearch's 4 GB data input limit, pipeline evaluation under a range of data usage scenarios indicates that overall performance generally does not improve, and in some cases dramatically declines, when more data are used for MR construction. Using reads from the single most read-abundant genotype for MR assembly, rather than using all available (population-wide) data, remains the recommended practice, regardless of Vsearch's ability to handle more data (Table 1).

### 2.2 Homolog variant detection

Despite the many advantages of GBS data, its reliability for reference-independent (or *de novo*) variant calling is compromised by the presence of homologous genomic regions. Whether the result of gene duplication (intragenomic homology) or polyploidization (homology across subgenomes), the existence of multiple copies of highly related but non-allelic sequences hampers reliable genotyping due to the challenge of separating such sequences into their respective loci (Dufresne *et al.*, 2014; Waples *et al.*, 2015). While the study of duplicated loci can shed light on fundamental evolutionary factors such as the adaptive potential of redundant genes and their role in the process of speciation (Madlung, 2013), paralogs and duplicated loci routinely confound population genomic studies, especially in polyploid species (Limborg *et al.*, 2016).

To address this fundamental issue and help users distinguish real allelic variation from artifactual polymorphisms due to homology (i.e. homolog variants), GBS-SNP-CROP now calculates for each called variant the mean allele depth ratio observed across all heterozygous individuals. Following the strategy described by McKinney *et al.* (2017), the deviation of this ratio from its expected value (1:1) is expressed as a *Z*-score, based on a binomial distribution with *P* = 0.5. Using these *Z*-scores, reported in a new column in the pipeline's final genotyping matrix, users can now identify and filter likely homolog variants. To test the informativeness of this new filter, SNPs and indels were called in populations of two different plant species, one diploid (*Berberis* × *ottawensis*) and one tetraploid (*Actinidia arguta*). Using a conservative threshold of |*Z*_i_| > 5 to declare likely homolog variants, the percentages of culled loci for the diploid and tetraploid species were 14.3% and 40.1%, respectively (Supplementary Table S1).

### 2.3 Improved accuracy

To assess the accuracy of GBS-SNP-CROP v.4.0 relative to the pipeline's initial release (v.1.0), we simulated a set of 150 bp single-end GBS reads with GBS-Pacecar. Across the 100 000 unique base GBS fragments simulated, 25 000 SNPs and 10 000 indels were induced, with no more than one variant per fragment. Approximately 60 million reads were generated across a population of 25 individuals, with read depth between 20-30x and sequencing error rate of 1.1%. As described in the GBS-Pacecar documentation, the details of all induced polymorphisms were recorded to enable downstream validation.

In addition to a significant improvement in speed, both Type I and Type II error rates are lower in v.4.0 than in the original version (Table 1). Indeed, the overall accuracy of the pipeline increased significantly from 65.4 to 84.0%, in large part due to its expanded indel functionality. As mentioned above, Table 1 also confirms that increased data usage for MR construction (e.g. multiple genotypes versus one genotype) results in higher error rates and poorer overall performance. Applying the same depth criteria for SNP genotyping, the Type I error for TASSEL-UNEAK (Lu *et al.*, 2013) was only slightly higher than that of GBS-SNP-CROP v.4.0, although it called less than one-tenth of the number of validated variants. UNEAK's Type II error, however, was enormous (92.5%), in part due to the pipeline's 64 bp read length requirement, leading to an overall accuracy of only 7.5%.

In the original release of GBS-SNP-CROP (Melo *et al.* 2016), it was observed that the sets of SNPs called by v.1.0 and UNEAK did not overlap completely. Such orthogonality begs the question, ‘Which set is right?’ What is notable about this simulation is that it shows that each pipeline calls correct variants but neither calls the complete set, lending credence to the idea of applying both to the same set of data. As shown in Supplementary Figure S2, however, the new version of GBS-SNP-CROP resolves this earlier issue of orthogonality. Through improved MR assembly, v.4.0 now detects all SNPs called by UNEAK, including those missed by v.1.0.

## 3 Conclusions

The GBS-SNP-CROP pipeline has proven to be a useful bioinformatics tool in the cost-effective genomic study of a wide range of plant and animal species; and updates since its initial release have expanded its functionality, improved its accuracy, and enhanced its overall performance. With the ability to handle variable-length single-end and paired-end reads, to detect both SNPs and indels, and to identify likely homolog variants, the most recent version of GBS-SNP-CROP (v.4.0) is a robust and versatile tool for variant calling in both model and non-model species.

## Supplementary Material

bty873_Supplementary_Data
