# Expanded functionality, increased accuracy, and enhanced speed in the *de novo* genotyping-by-sequencing pipeline GBS-SNP-CROP

**DOI:** 10.1093/bioinformatics/bty1073

**Published:** 2019-02-06

**Authors:** Arthur T O Melo, Iago Hale

*Bioinformatics* (2019) doi: 10.1093/bioinformatics/bty873, **35**, 1783–1785.

In the original article, there was an error in the formatting of Table 1.

**Table 1.** Comparative summary of GBS-SNP-CROP v.4.0 performance, based on a set of simulated data from GBS-Pacecar

Pipeline^a^	MR geno^b^	Time (min)^c^	Variants called^d^	Type I error^e^	Type II error^f^	Accuracy^g^
UNEAK	NA	8.5	2642	0.9%	92.5%	7.5%
GSC v.1.0	1	370.8	23 395	1.3%	34.1%	65.4%
GSC v.4.0	1	121.7	29 738	0.6%	15.6%	84.0%
	5	156.9	26 885	0.6%	23.6%	76.0%
	10	171.5	26 854	0.5%	23.7%	76.1%
	15	179.1	26 897	0.5%	23.6%	76.1%
	20	183.0	26 892	0.5%	23.6%	76.1%
	25	163.2	26 901	0.5%	23.5%	76.2%

*Note*: In total, 25 000 SNPs and 10 000 indels were simulated across a genomic space of 100 000 GBS fragments. A total of 60 002 165 single-end reads were simulated for a population of 25 individuals (average of 2.4 million reads per genotype), with a sequencing error rate of 1.1%. See Supplementary Table S1 for more details

a UNEAK = TASSEL-UNEAK; GSC = GBS-SNP-CROP.

b The number of genotypes used for mock reference (MR) assembly.

c Computation time (minutes) required to run the full analysis on a Unix workstation with 16 GB RAM and a 2.6 GHz Dual Intel processor.

d Number of variants called by a pipeline (Note: a total of 35 000 variants were simulated, consisting of 25 000 SNPs and 10 000 indels).

e Percentage of called variants that could not be validated (false positives).

f Percentage of true, simulated variants that were not detected by the pipeline.

g Overall accuracy: 100 * [number of validated variants/(total number of simulated variants + number of non-validated variants)].

This has been corrected and the corrected table appears below.

