# Supplementary material for: Expanded functionality, increased accuracy, and enhanced speed in the de novo genotyping-by-sequencing pipeline GBS-SNP-CROP
Source: Bioinformatics. 2018 Oct 15;35(10):1783–5. doi: 10.1093/bioinformatics/bty873 (PMC6513162; doi:10.1093/bioinformatics/bty873)
Supplement: bty873_Supplementary_Data [file bty873_supplementary_data.docx]

Expanded functionality, increased accuracy, and enhanced speed in the *de novo* genotyping-by-sequencing pipeline GBS-SNP-CROP

Arthur T O Melo^1^ and Iago Hale^1*^

^1^ University of New Hampshire, Department of Agriculture, Nutrition, and Food Systems, Durham, NH, USA.

**Supplementary Data**

**Fig. S1**

**Fig. S2**

**Table S1**

**Table S2**


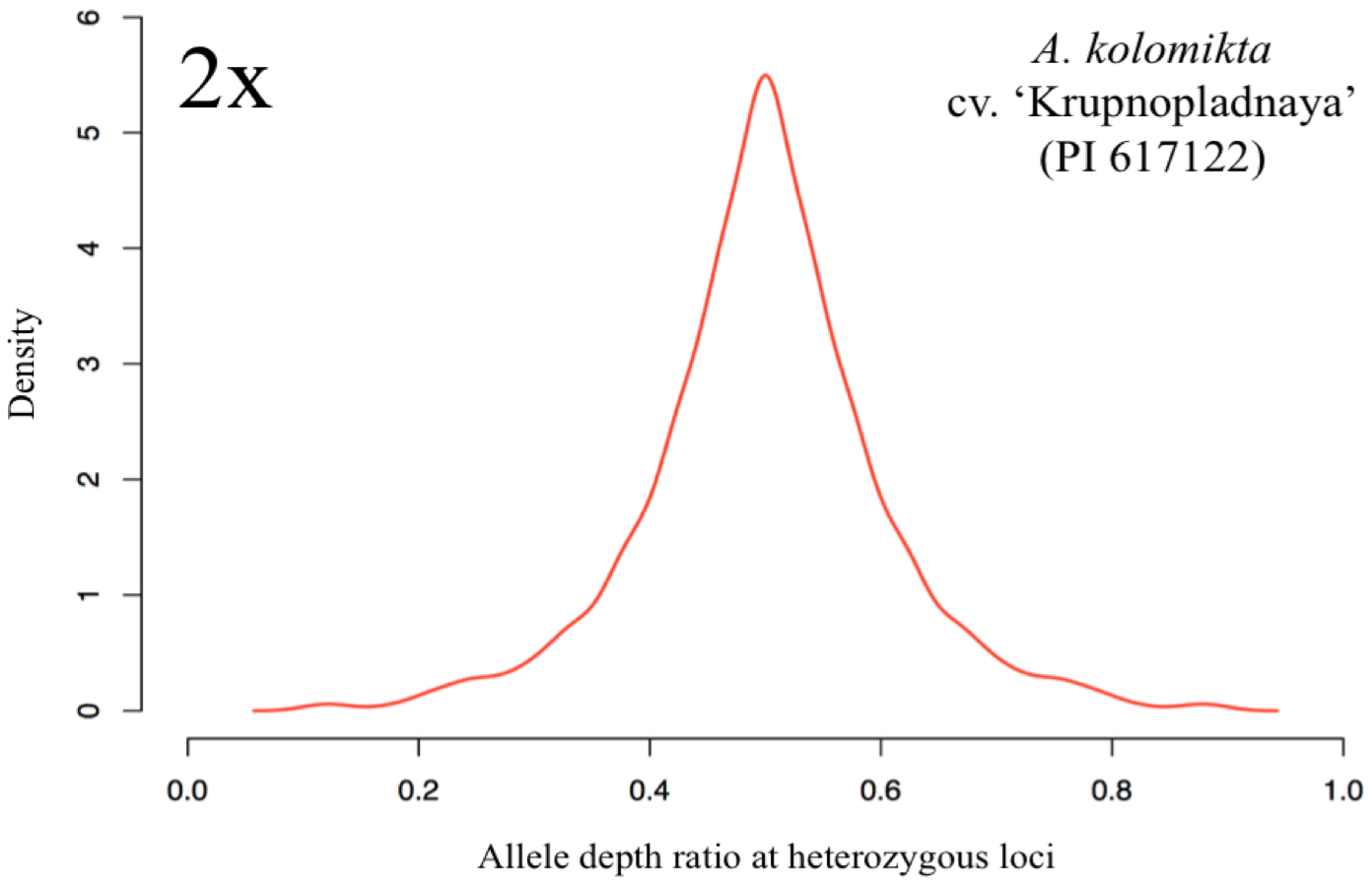


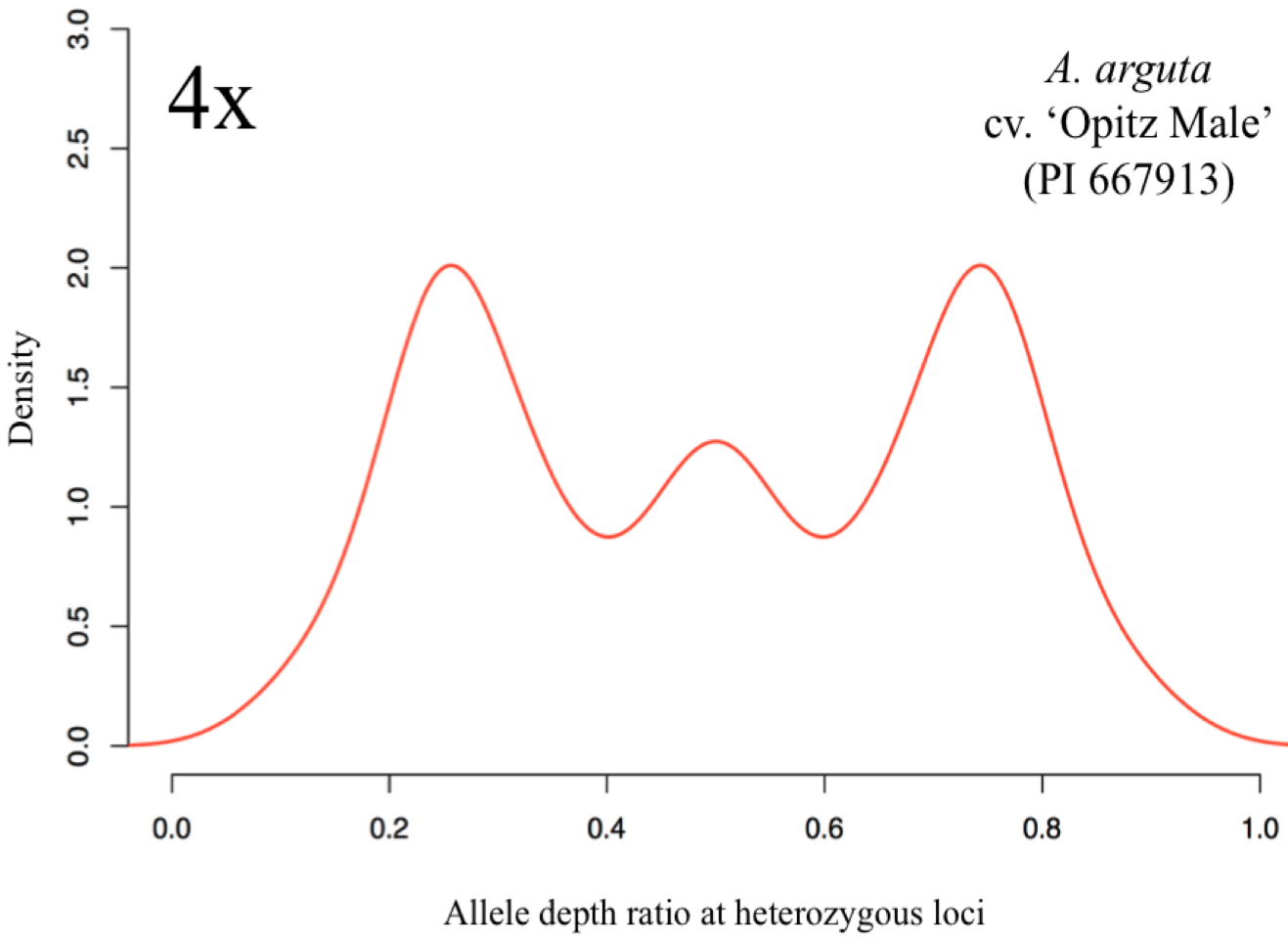


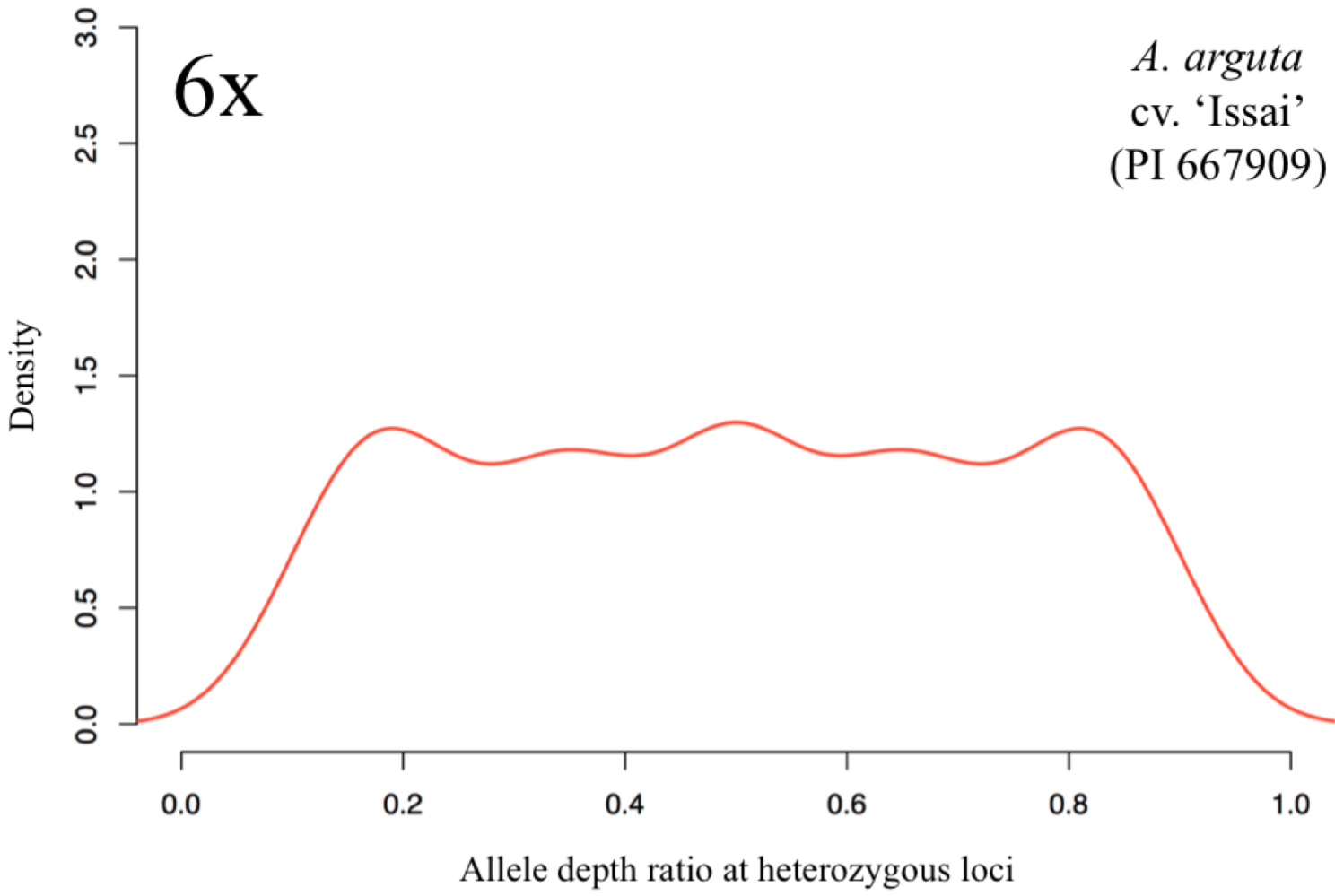


**Fig. S1** The new script PloidyVisualizer.pl enables GBS-SNP-CROP users to easily generate distributions of allele depth ratios at heterozygous loci as a means of ascertaining the ploidy levels of individual genotypes. For a diploid (2x) accession (top), the distribution should exhibit a single mode at 0.5. For a triploid (3x), there should be two modes, at 0.33 and 0.67; for a tetraploid (4x), three modes, at 0.25, 0.5 and 0.75 (middle); and so on, up to hexaploids (bottom) and beyond. See GBS-SNP-CROP User Manual for details.

**
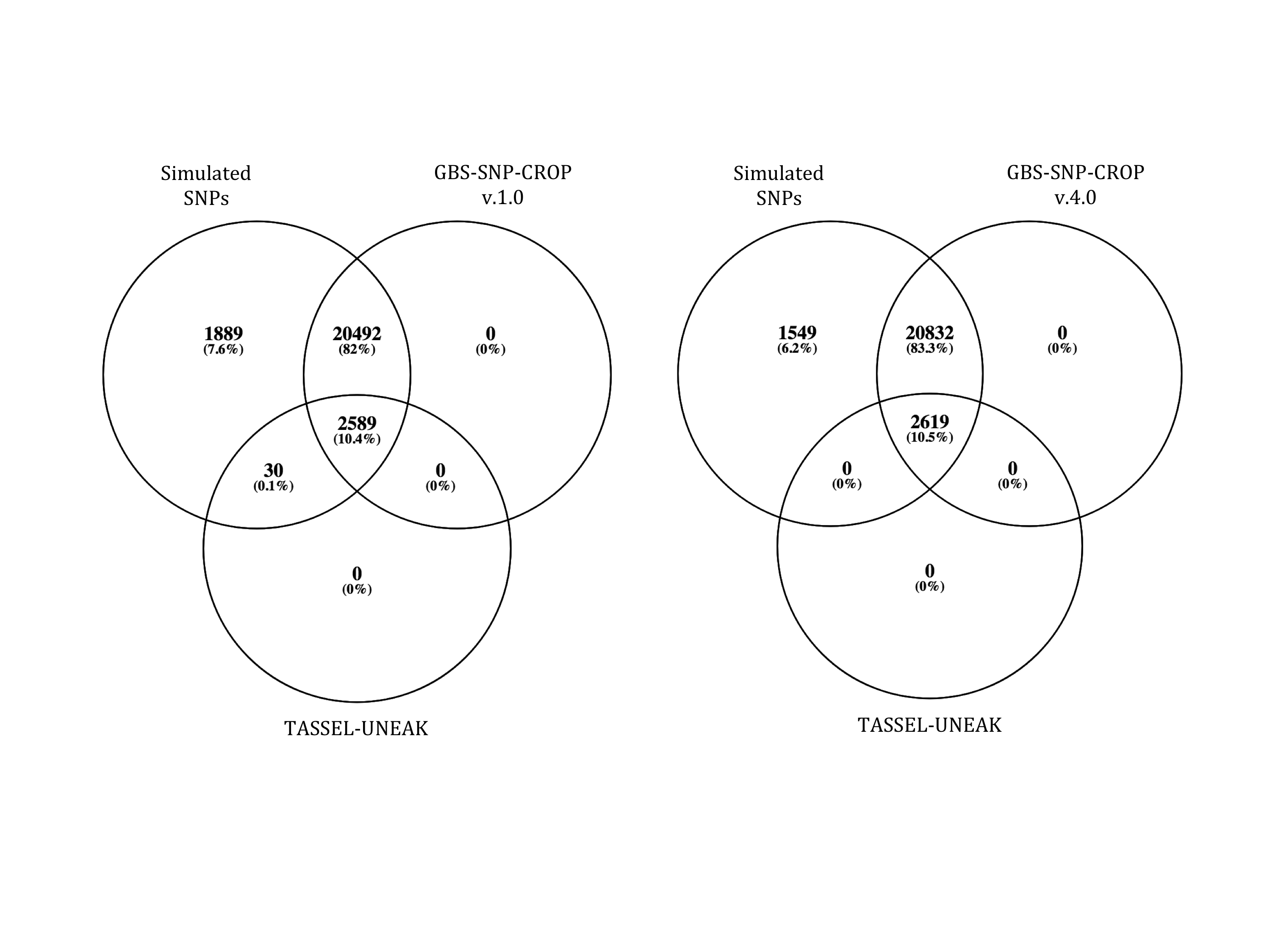
**

**Fig. S2** Venn diagrams of the sets of validated SNPs called between TASSEL-UNEAK and versions 1.0 (left) and 4.0 (right) of GBS-SNP-CROP, using 150 bp single-end reads simulated by GBS-Pacecar (command: perl GBS-Pacecar.pl -n 100000 -snps 0.25 -indels 0.1 -minl 32 -maxl 150 -g 25 -mind 20 -maxd 30 -b barcodeID.txt). The non-overlap, or orthogonality, observed between the GBS-SNP-CROP v.1.0 and UNEAK sets of SNPs (A) is resolved in GBS-SNP-CROP v.4.0 (B), as a result of to optimized MR construction.

**Table S1** Comparative summary of GBS-SNP-CROP v.4.0 performance, based on a set of simulated data from GBS-Pacecar. 25,000 SNPs and 10,000 indels were simulated across a genomic space of 100,000 GBS fragments. A total of 60,002,165 single-end reads were simulated for a population of 25 individuals (average of 2.4 million reads per genotype), with a sequencing error rate of 1.1%.

| **Pipeline** | **MR geno^a^** | **Mean cluster length (bp)^b^** | **MR length (Mbp)^c^** | **Time (min)^d^** | **Var type^e^** | **Variants called^f^** | **Variants validated^g^** | **Variants validated (%)** | **Mean**  **depth^h^** | **Type I**  **error (%)^i^** | **Type II**  **error**  **(%)^j^** | **Accuracy**  **(%)^k^** |
| --- | --- | --- | --- | --- | --- | --- | --- | --- | --- | --- | --- | --- |
| TASSEL-UNEAK | N/A | N/A | N/A | 8.5 | SNP | 2,642 | 2,619 | 99.1 | 6.6 | 0.9 | 89.5 | 10.5 |
|  |  |  |  |  | Indel | 0 | 0 | -- | -- | -- | 100.0 | -- |
|  |  |  |  |  | Both | 2,642 | 2,619 | 99.1 | 6.6 | 0.9 | 92.5 | 7.5 |
| GBS-SNP-CROP v.1.0 | 1 | 90.48 | 9.28 | 370.8 | SNP | 23,395 | 23,081 | 98.7 | 23.6 | 1.3 | 7.7 | 91.2 |
|  |  |  |  |  | Indel | 0 | 0 | -- | -- | -- | 100.0 | -- |
|  |  |  |  |  | Both | 23,395 | 23,081 | 99.7 | 23.6 | 1.3 | 34.1 | 65.4 |
| GBS-SNP-CROP v.4.0 | 1 | 90.10 | 9.36 | 121.7 | SNP | 23,625 | 23,451 | 99.3 | 23.6 | 0.7 | 6.2 | 93.2 |
|  |  |  |  |  | Indel | 6,113 | 6,103 | 99.8 | 23.2 | 0.2 | 39.0 | 61.0 |
|  |  |  |  |  | Both | 29,738 | 29,554 | 99.4 | 23.5 | 0.6 | 15.6 | 84.0 |
|  |  |  |  |  |  |  |  |  |  |  |  |  |
| GBS-SNP-CROP v.4.0 | 5 | 88.65 | 9.71 | 156.9 | Both | 26,885 | 26,732 | 99.4 | 23.6 | 0.6 | 23.6 | 76.0 |
|  | 10 | 88.59 | 9.72 | 171.5 |  | 26,854 | 26,720 | 99.5 | 23.6 | 0.5 | 23.7 | 76.1 |
|  | 15 | 88.61 | 9.72 | 179.1 |  | 26,897 | 26,754 | 99.5 | 23.6 | 0.5 | 23.6 | 76.1 |
|  | 20 | 88.64 | 9.72 | 183.0 |  | 26,892 | 26,746 | 99.5 | 23.6 | 0.5 | 23.6 | 76.1 |
|  | 25 | 88.71 | 9.71 | 163.2 |  | 26,901 | 26,760 | 99.5 | 23.6 | 0.5 | 23.5 | 76.2 |

**^a^** The number of genotypes used for mock reference (MR) assembly

**^b^** Average length of the MR clusters (bp)

^c^ Total length of the assembled MR (Mbp)

**^d^** Computation time (minutes) required to run the full analysis on a Unix workstation with 16 GB RAM and a 2.6 GHz Dual Intel processor

^e^ Type of variant called (Both = SNP + indel)

^f^ Number of variants called by a pipeline (Note: A total of 35,000 variants were simulated, consisting of 25,000 SNPs and 10,000 indels)

^g^ Number of validated variants, where a called variant is considered validated if its type and position agree with an induced polymorphism in the simulated data

**^h^** Mean read depth for all called variants across the entire population

^i^ Percentage of called variants that could not be validated (false positives)

^j^ Percentage of true, simulated variants that were not detected by the pipeline

^k^ Overall accuracy: 100 * [ number of validated variants / ( total number of simulated variants + number of non-validated variants ) ]

**Table S2** Comparative summary statistics from GBS-SNP-CROP v.4.0 for two populations of 25 individuals each, one diploid (*Berberis* ×*ottawensis*) and one allotetraploid (*Actinidia arguta*). Compared to the diploid case, widespread homology between the subgenomes of *A. arguta* results in: 1) Higher mean depth due to homolog pile-up, and 2) A higher proportion of likely homolog variants (i.e. false, artifactual polymorphisms between homologs that should be culled). GBS-SNP-CROP users can now inspect and filter such variants, based on the homolog variant detection statistic reported in the pipeline's final genotyping matrix.

| **Species** | **Ploidy** | **Time (min)** | **Var type** | **Variants called** | **Mean**  **depth** | **Homolog variants (%)^b^** |
| --- | --- | --- | --- | --- | --- | --- |
| *Berberis* ×*ottawensis* | 2x | 79.1 | SNP | 15,501 | 47.4 | 14.4 |
|  |  |  | Indel | 1,817 | 45.0 | 13.4 |
|  |  |  | Both | 17,318 | 47. 1 | 14.3 |
| *Actinidia arguta* | 4x | 141.6 | SNP | 16,932 | 66.0 | 40.6 |
|  |  |  | Indel | 1,207 | 56.7 | 33.6 |
|  |  |  | Both | 18,139 | 65.4 | 40.1 |

**^a^** Percentage of called variants that are likely polymorphisms between homologs (i.e. artifactual variants) based on a deviation of |Z_i_| > 5, as proposed by McKinney et al. (2017).
